# Optimized Quantum Drude Oscillators for Atomic and Molecular Response Properties

**DOI:** 10.1021/acs.jpclett.3c01221

**Published:** 2023-06-29

**Authors:** Szabolcs Góger, Almaz Khabibrakhmanov, Ornella Vaccarelli, Dmitry V. Fedorov, Alexandre Tkatchenko

**Affiliations:** Department of Physics and Materials Science, University of Luxembourg, L-1511 Luxembourg City, Luxembourg

## Abstract

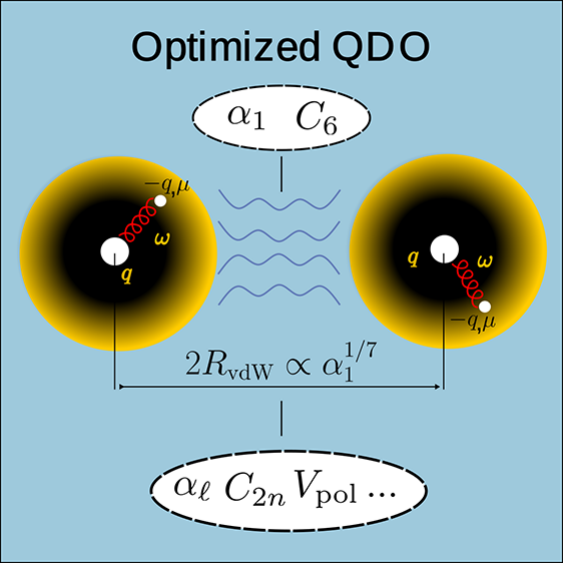

The quantum Drude oscillator (QDO) is an efficient yet accurate coarse-grained approach that has been widely used to model electronic and optical response properties of atoms and molecules as well as polarization and dispersion interactions between them. Three effective parameters (frequency, mass, and charge) fully characterize the QDO Hamiltonian and are adjusted to reproduce response properties. However, the soaring success of *coupled* QDOs for many-atom systems remains fundamentally unexplained, and the optimal mapping between atoms/molecules and oscillators has not been established. Here we present an optimized parametrization (OQDO) where the parameters are fixed by using only dipolar properties. For the periodic table of elements as well as small molecules, our model accurately reproduces atomic (spatial) polarization potentials and multipolar dispersion coefficients, elucidating the high promise of the presented model in the development of next-generation quantum-mechanical force fields for (bio)molecular simulations.

The development of predictive model Hamiltonians that can describe various properties of realistic molecules and materials is a cornerstone of modern physics^1^ and chemistry.^2^ The quantum Drude oscillator (QDO) is arguably the most powerful Hamiltonian (see the Supporting Information) for accurate and efficient modeling of atomic and molecular response.^1,3−10^ Within the coarse-grained QDO model, the response of valence electrons is described *via* a quasi-particle *drudon* with a negative charge −*q* and mass μ, harmonically bound to a positively charged pseudonucleus of charge *q* with a characteristic frequency ω. The many-body extension of the QDO model (the *coupled* QDO model) has been widely employed to study both molecules and materials, including their electronic^11,12^ and optical^13^ properties, polarization,^14,15^ dispersion,^7,14,16−24^ and exchange^25−27^ interactions, as well as a wealth of nonadditive field effects in quantum mechanics^23,28^ and quantum electrodynamics.^24,29^ Coupled QDOs are also extensively used in the development of van der Waals (vdW) density functionals,^18,30,31^ quantum mechanical^1,6^ and polarizable force fields,^32−36^ as well as recent machine learning force fields.^37,38^ Despite such a wide applicability of the coupled QDO model, its success in describing real atoms remains fundamentally unexplained, and the optimal mapping between atoms and oscillators has not been established. In this Letter, we develop an optimized parametrization (OQDO) in which the parameters are fixed by using only the well-known atomic dipolar properties. Remarkably, the OQDO reproduces spatial atomic polarization potentials and atomic multipolar dispersion coefficients. Our OQDO model for atoms and small molecules also paves the way to the development of next-generation quantum-mechanical force fields for (bio)molecular simulations.

The three parameters {*q*, μ, ω} fully define the QDO, and three atomic response properties could be chosen to fix them, meaning that the choice of QDO parameters is not unique. In addition, all QDO response properties, multipolar polarizabilities and dispersion coefficients, are uniquely fixed by the three parameters *via* closed-form relations.^6^ The static dipole polarizability of a QDO, α_1_ = *q*^2^/*μω*^2^, conveniently combines all three parameters, and it is natural to set this expression to the reference atomic α_1_. The QDO expression for the dipole–dipole dispersion coefficient  is identical to the London formula and allows to fix ω if the reference atomic values of *C*_6_ and α_1_ are given. Since α_1_ and *C*_6_ are accurately known for all elements in the periodic table,^39−41^ they form a baseline for the QDO parametrization. However, one more condition is required to obtain {*q*, μ, ω}, for which different constraints can be imposed. A reasonable idea is to fix *q* = 1 au since a QDO should reproduce the response of electrons. This results in the fixed-charge QDO (FQDO):
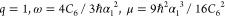
1However, fixing *q* and using QDO recursion relations for high-order response usually yields large errors in the multipolar response properties (see Figure 3 and refs (42 and 43)). A more rigorous approach was suggested by Jones et al.^6^ by employing the dipole–quadrupole dispersion coefficient *C*_8_. The mapping {α_1_, *C*_6_, *C*_8_} → {*q*, μ, ω} yields the Jones QDO (JQDO) parametrization scheme:
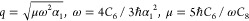
2The JQDO approach improves the multipolar response over the FQDO model, while simulations using the coupled JQDO model captured many remarkable properties of bulk water and its surface.^35,44^ However, the *C*_8_ dispersion coefficient is not directly measurable, and accurate *ab initio* calculations of quadrupole (α_2_) and octupole (α_3_) polarizabilities and *C*_8_–*C*_10_ dispersion coefficients are currently technically feasible only for closed-shell species (noble-gas atoms and small molecules) or alkali and alkaline-earth atoms with *s* valence shells.^45−48^ For other open-shell atoms (containing *p*, *d*, or *f* valence shells), convergence of quantum-chemical response calculations becomes a technical hurdle^49^ (see also additional discussion in the Supporting Information). Thus, using higher-order atomic response properties does not lead to a parametrization that would be universally applicable across the periodic table as well as for small molecules.

Here, we introduce an optimized QDO parametrization (OQDO), where we effectively map dipolar atomic quantities {α_1_, *C*_6_} to the oscillator parameters. The third parameter is fixed by using the force balance equation for vdW-bonded dimers derived recently.^25−27^ Two equations for *q* and ω follow the JQDO scheme, whereas the third one is replaced with a transcendental equation for a product *μω* to be solved numerically (*vide infra*)
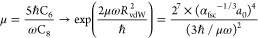
3where α_fsc_ = *e*^2^/4*πε*_0_*ℏc* is the fine-structure constant and *a*_0_ is Bohr’s radius. The vdW radius (*R*_vdW_) is calculated *via* the universal formula connecting it with the dipole polarizability
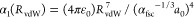
4as established in ref (27) for atoms in the periodic table. Comparing it with its counterpart
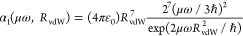
5which was obtained within the QDO model,^25,26^ delivers eq 3 to determine μ from *μω*.

The QDO Hamiltonian effectively captures the integrated atomic response. However, when modeling molecules or solids, coupled QDOs must properly describe noncovalent interactions between atoms. Considering two fragments *i* and *j* and using interatomic perturbation theory,^50,51^ the interaction energy can be written as the integrated product of the electron density of moiety *i* with the electric potential generated by moiety *j*:^50,52^
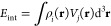
6This formula is valid for all noncovalent interactions: electrostatics, induction, exchange-repulsion, and dispersion. Its validity is evident for the former two cases,^50,52^ and it was shown that exchange^53^ and dispersion^54−56^ interactions can be represented using the form of eq 6 with ρ and *V* being *effective* quantities different from free-atom counterparts. The argument that dispersion interactions can be written using eq 6 goes back to Feynman’s consideration of molecular forces,^54^ which was further elaborated by Hunt^55^ with a focus on dispersion forces and finally extended to dispersion energies with a demonstration of its validity for real molecules and materials.^57,58^

Response properties are given by variations of *E*_int_ as
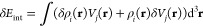
7For an external electric field **E**, which can also model the effect of environment, *δρ*(**r**) = ρ_*E*_(**r**) – ρ(**r**), where ρ_*E*_(**r**) is the electron density under the external field. Then the dominant contribution to *δV*_j_(**r**) is generated by the corresponding *δρ*_*j*_(**r′**) via the polarization potential^59^
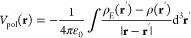
8which describes the change in the electrostatic potential of the system due to the polarization of its charge density by the presence of another moiety (an electric field in this case). For the QDO in a uniform electric field, the integral in eq 8 can be evaluated analytically as
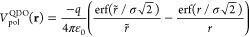
9where  is the field-induced oscillator coordinate and  is the QDO spread.^60^ In the Supporting Information, we present *V*_pol_^QDO^(**r**) in comparison to *V*_pol_(**r**) calculated for 21 atoms (between H–Ca and Kr) within hybrid density functional theory DFT-PBE0^61−65^ shown to yield a highly accurate description of electronic response^66^ comparable to coupled-cluster calculations (see the Supporting Information). Here, we remark that the strength of the electric field was chosen individually for each element depending on its reference static dipole polarizability^39,41^ so that the field-induced dipole moment is set as **d** = α_1_**E** = 0.01 au for all atoms.^67^

Before comparing *V*_pol_(**r**) for real atoms with different QDO flavors, it is instructive to consider which atomic properties can be faithfully captured by a QDO. First, the QDO does not aim to describe static properties of the atomic electron density but rather its response under applied static and fluctuating fields, as demonstrated also by the insets in Figure 1a,b. The electrostatic potential (ESP) of a QDO is given by , so the charge *q* determines its magnitude. This explains why *V*_el_^FQDO^ yields good agreement with *V*_el_^DFT^ for hydrogen. However, the QDO model does not describe *V*_el_ for many-electron atoms because *q* ≈ 1 au, while the ESP of atoms scales nonlinearly with *Z* (see the example of carbon in the inset of Figure 1b). Second, the harmonic response captured by a QDO model should be sufficient to accurately describe integrated electronic displacements induced by weak fields. However, it is much less clear how well different QDO parametrizations perform for distributed polarization potentials described by eq 8 for many-electron systems, given the analytical form of *V*_pol_^QDO^(**r**) in eq 9. To answer this question, in Figure 1 we compare the *V*_pol_ curves of real atoms and *V*_pol_^QDO^(**r**) employing the three QDO models discussed above. We used the accurate *ab initio* reference data on α_1_ and *C*_6_(39,41,68) to parametrize FQDO and OQDO. When available, we also used the analogous data on *C*_8_(45−47) to parametrize the JQDO model. We observe that the OQDO model is able to reproduce the full range of the polarization potential of real atoms with a reasonable accuracy, showing significantly better agreement with the DFT-PBE0 results than FQDO and JQDO. To quantify this, for each atom, we calculated the root–mean–squared-error (RMSE) of the three QDO curves with respect to the PBE0 reference curves and normalized the RMSE using the equilibrium depth of the PBE0 curve. The OQDO flavor has an error of 8.9% when averaged over 21 atoms, whereas JQDO and FQDO yield average errors of 13.2% and 15.4%, respectively. We also emphasize that the predictions of the OQDO model remain accurate for many-electron atoms such as noble gases and alkali metals. It is especially reassuring that the OQDO model reproduces the nonlinear *V*_pol_(**r**) curves obtained from DFT calculations without any adjustments. In fact, the performance of the OQDO is sensitive to variations in the QDO parameters (solutions A or B in Figure 1), so the satisfactory agreement shows that the chosen OQDO(A) model accurately describes real atoms. The significant differences between the predictions of various parametrizations for *V*_pol_(**r**) underline the importance of optimal mapping between atomic response properties and QDO parameters.

**Figure 1:**
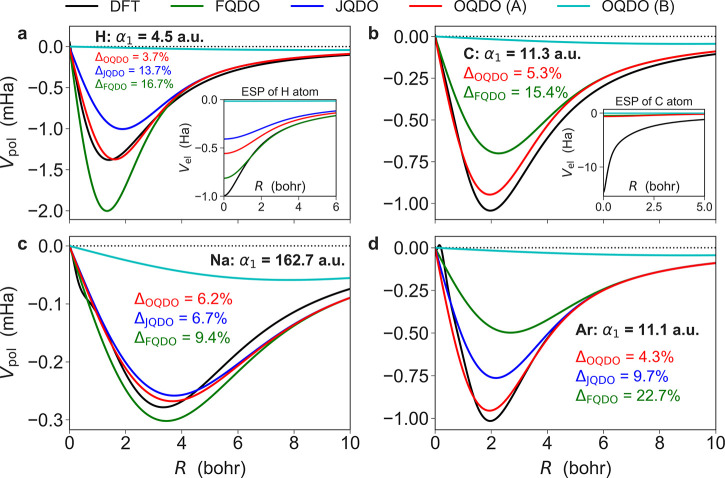
Polarization potential curves *V*_pol_(**r**) calculated with DFT-PBE0 and various QDO parametrizations for (a) hydrogen, (b) carbon (no JQDO values are available), (c) sodium, and (d) argon atoms. The FQDO and JQDO parametrization schemes are described in eqs 1 and 2, respectively. OQDO(A) and OQDO(B) correspond to the two solutions of the transcendental equation given by eq 3. In all cases, the direction along the applied field was chosen for the plots. The reference values for dipole polarizability α_1_ are shown for each element. The numerical values of the normalized root–mean–square error (Δ) are displayed for the three QDO flavors. For hydrogen and carbon atoms, the unperturbed electrostatic potentials (ESP) *V*_el_(**r**) are shown as insets, indicating that a QDO captures the response of atomic electron density but not the static potential itself.

We discuss now the technical aspects of deriving the two solutions of the OQDO model ( labeled as OQDO(A) and OQDO(B) in Figure 1 and the Supporting Information) and their connection to real atoms. The starting point is eq 4 that connects the atomic vdW radius and its dipole polarizability. Within the QDO model, eq 4 can be written as follows:^26^
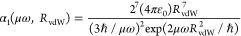
10The OQDO parametrization imposes that the product *μω* in eq 10 delivers the same *R*_vdW_ as from eq 4, for α_1_(*μω*, *R*_vdW_) = α_1_(*R*_vdW_). For simplicity, we rewrite eq 10 in terms of the dimensionless variable *x* as
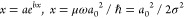
11with the dimensionless coefficients *a* and *b* given by
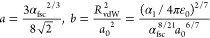
12where we used eq 4 to express *R*_vdW_ in terms of α_1_. For all elements in the periodic table, we found that eq 11 has two solutions, A and B. This is illustrated by the inset of Figure 2b for the case of Ar. It is instructive to consider that eq 11 has one solution when the polarizability of an atom is equal to the critical value:
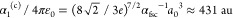
13which is greater than the largest atomic dipole polarizability (α_1_ ≈ 400 au) of Cs.^41^ The existence of two solutions extends beyond the employed QDO model. We obtained an analogous result by using the Tang-Toennies potential^69^ with the repulsive interaction treated by the Born-Mayer form (see the Supporting Information).

**Figure 2:**
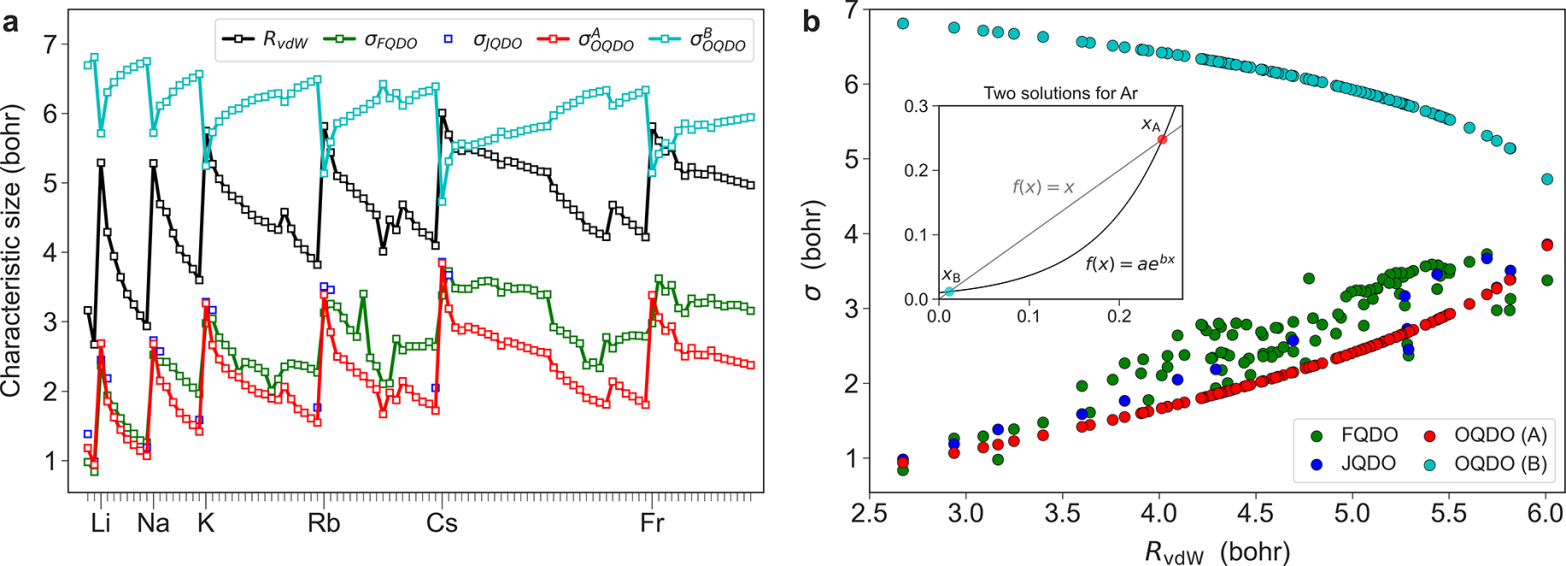
(a) Periodic variations of the QDO length  with the atomic number for the three different parametrizations, as compared to the atomic vdW radii (*R*_vdW_), which are evaluated *via*eq 4 using the reference atomic polarizabilities.^39^ (b) Correlation between *R*_vdW_ and σ (within the three QDO parametrizations) for 102 elements in the periodic table. The schemes of OQDO(A) and OQDO(B) correspond to two solutions of eq 11, as illustrated using the example of Ar in the inset.

Since the frequency of the OQDO is fixed by the second condition of eqs 1 and 2, solutions A and B for the product *μω* differ both in mass and charge, yielding quite different results. First, *V*_pol_^QDO^(**r**) constructed from solution B does not resemble DFT potentials, while A is in good agreement with them (Figure 1). Second, the overlap integral  between two QDOs at their equilibrium distance *R*_eq_ = 2*R*_vdW_ is significantly larger for solution B, which violates the initial assumption used to derive eq 10 that *S* is small at *R*_eq_.^26^ Third, the QDO length σ constructed from solution A follows the same periodic trend as the atomic vdW radii, whereas solution B does not seem to correlate well (Figure 2a). Therefore, throughout this work we refer to solution A as the optimized parametrization. For 102 atoms, the full set of QDO parameters corresponding to both solutions A and B is presented in the Supporting Information together with the reference values of {α_1_, *C*_6_}. Another noteworthy property of the OQDO model (see Figure 2b) is a clear correlation between the QDO length (model quantity) and the atomic vdW radius (physical observable). In fact, these quantities should be connected *via* the dipole polarizability.^26,60^ This property is not captured well by either the FQDO or the JQDO models.

For practical calculations of the vdW energy and constructing predictive force fields, the multipolar contributions associated with the *C*_8_ and *C*_10_ coefficients can become relevant.^42,43,48^ The available reference data for higher-order molecular dispersion coefficients have significant uncertainties. Our careful examination of the literature reporting the reference values of *C*_8_ and *C*_10_ (see refs (45−48). and references therein) identifies uncertainties of up to 20% for the reference *C*_8_ and *C*_10_ values. Within the QDO formalism, it is straightforward to evaluate these coefficients using closed-form expressions derived by Jones et al.^6^ In Figure 3, we present the predictions of *C*_8_ and *C*_10_ by FQDO and OQDO models as compared to accurate reference values compiled from the literature^45−48^ for a set of 16 atoms (including alkali and alkaline-earth metals and noble gases) and 12 small molecules. Overall, our results show that the OQDO parametrization improves the dispersion coefficients compared to the FQDO one, reducing the MARE from 31% to 25% for *C*_8_ and from 68% to 33% for *C*_10_ when averaged over all 28 (26 for *C*_10_) systems. The OQDO model consistently surpasses the FQDO in accuracy for all systems except for alkaline-earth metals where the FQDO gives more accurate results. Moreover, Figure 3 shows that the deviations of OQDO dispersion coefficients from the reference values are consistent in terms of their sign and magnitude. Namely, for the majority of systems, FQDO underestimates *C*_8_ and *C*_10_, but roughly for one-third of them, the dispersion coefficients are overestimated. The maximal errors of FQDO are observed for Xe in the case of both *C*_8_ (66%) and *C*_10_ (190%). In contrast, the OQDO consistently underestimates both dispersion coefficients for all systems, except for *C*_8_ of Li as well as *C*_10_ of Li and Cs. The maximum errors of the OQDO are observed for CO_2_ (46%) in the case of *C*_8_ and CO (58%) in the case of *C*_10_, which are significantly smaller than maximum errors of the FQDO. The consistency of the OQDO errors allows a straightforward rescaling of dispersion coefficients: with our best rescaling factors, 1.3 for *C*_8_ and 1.5 for *C*_10_, one can decrease the MARE of the OQDO to 15% and 22%, respectively, which is commensurate with the uncertainty of the reference molecular *C*_8_ and *C*_10_ values. A more detailed analysis of the dispersion coefficients (including JQDO and OQDO models as well as their scaled versions) can be found in the Supporting Information, where we also discuss static polarizabilities α_2_ and α_3_. The latter becomes less important in the QDO approach where the dispersion coefficients, determining the dispersion energy, are directly expressed in terms of QDO parameters. It is important to mention that the effects of three-body interactions are captured by the OQDO scheme on an equal footing with the JQDO scheme. The accuracy in determination of *C*_6_ and *C*_9_ coefficients is known to be comparable.^70^ In ref (6), it was shown that within the QDO model the leading three-body dispersion coefficient is given by *C*_9_ = α_1_*C*_6_/4. Thus, with the same reference α_1_ and *C*_6_, there is no difference between the JQDO and the OQDO parametrizations.

**Figure 3:**
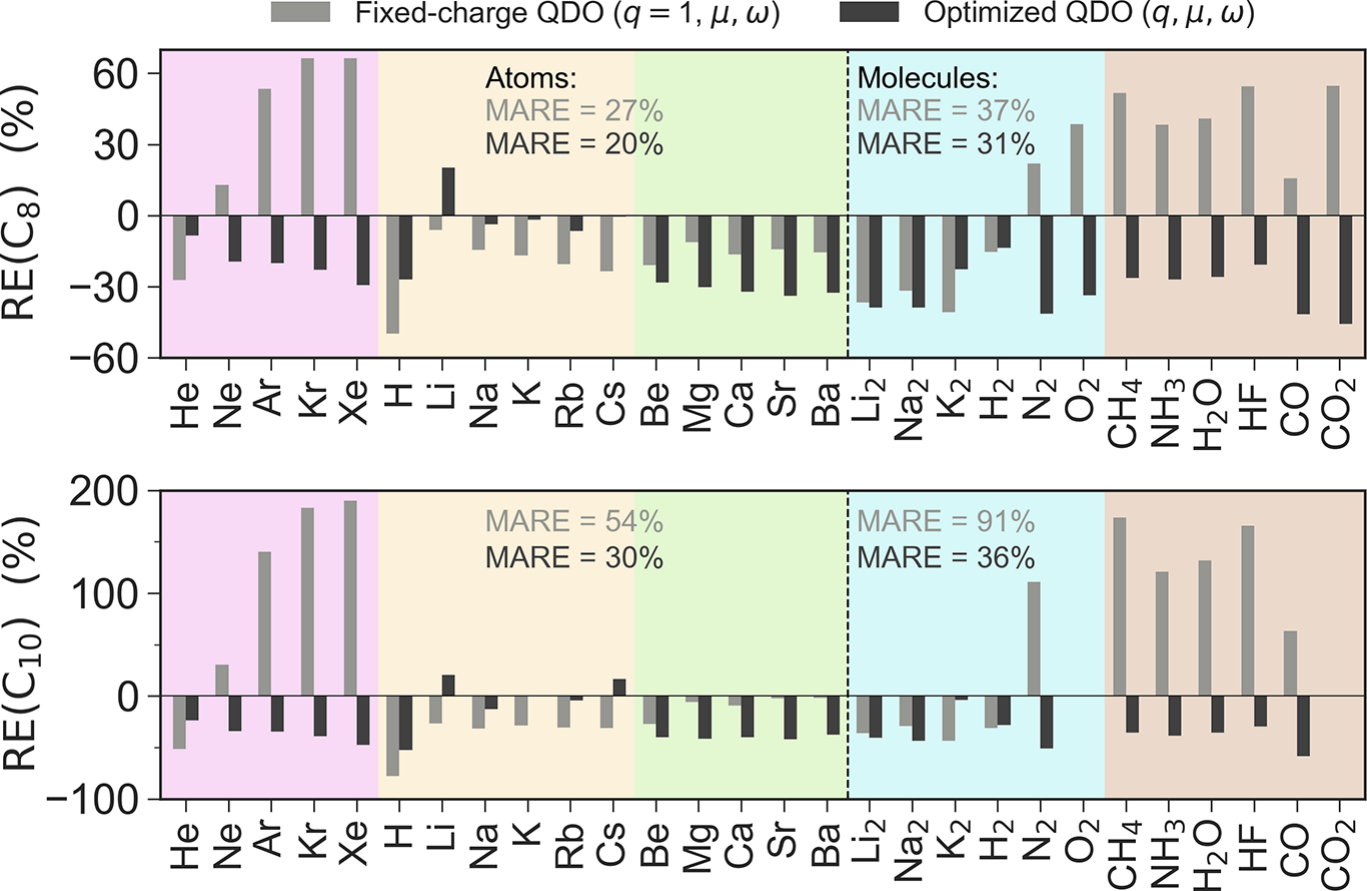
Multipolar dispersion coefficients *C*_8_ and *C*_10_ as predicted by the FQDO (*q* = 1) and OQDO models. Relative error RE = (*C*_*j*_–*C*_*j*_^ref^)/*C*_*j*_^ref^ with respect to *ab initio* reference data^45−48^ is plotted. For the two models, numerical values of mean absolute relative errors (MARE) are evaluated separately for atoms and molecules. In the cases of O_2_ and CO_2_, no reliable *ab initio* reference data for *C*_10_ could be found.

We presented the OQDO model based on robust parametrization that solely employs dipolar α_1_ and *C*_6_, accurately known for all atoms in the periodic table. The new parametrization scheme dispenses the need for reference higher-order dispersion coefficients and delivers accurate polarization potentials, thereby improving the description of noncovalent interactions at short distances keeping the accuracy of the JQDO model for large distances due to the proper dipolar response. The key point of the proposed parametrization is employing the relation between the dipole polarizability and the vdW radius, both being integrated quantities with many-electron effects included. Thus, the OQDO scheme serves as an optimized and efficient mapping between atoms/molecules and oscillators, which substantially advances our ability to model a wide range of response properties of molecules and materials, also paving the way to develop next-generation quantum-mechanical force fields for (bio)molecular simulations.

## Supporting Information Available

Polarization potentials for 21 chemical elements, OQDO parameters, dispersion coefficients and multipole polarizabilities, more detailed discussions and computational details (PDF)
