# Supplementary material for: Optimized Quantum Drude Oscillators for Atomic and Molecular Response Properties
Source: J Phys Chem Lett. 2023 Jun 29;14(27):6217–23. doi: 10.1021/acs.jpclett.3c01221 (PMC10351863; doi:10.1021/acs.jpclett.3c01221)
Supplement: Supplementary file 1 — jz3c01221_si_001.pdf [file jz3c01221_si_001.pdf]

Supporting Information: Optimized Quantum Drude Oscillators for Atomic and Molecular Response Properties

Szabolcs Góger, Almaz Khabibrakhmanov, Ornella Vaccarelli, Dmitry V. Fedorov, and Alexandre Tkatchenko*

Department of Physics and Materials Science, University of Luxembourg, L-1511 Luxembourg City, Luxembourg

E-mail: alexandre.tkatchenko@uni.lu

Brief derivation of the QDO formalism

Within the quantum Drude oscillator (QDO) model, the response of all valence electrons is represented by a single quasiparticle (*drudon*). The Hamiltonian of a single QDO is given by the well-known harmonic oscillator[1]

$$\hat{\mathcal{H}}_0 = -\frac{\hbar^2}{2\mu}\,\boldsymbol{\nabla}_{\mathbf{r}}^2 \;+\; \frac{1}{2}\,\mu\omega^2\mathbf{r}^2 \;.$$

The corresponding eigenvalues and eigenfunctions, respectively, are given by

$$E_{\{n_x n_y n_z\}} = \hbar\omega\left(n_x + n_y + n_z + \frac{3}{2}\right) \;,\quad E_{n_x} = \hbar\omega\left(n_x + \frac{1}{2}\right) \;,\quad n_x = 0, 1, 2, ...$$

$$\Psi_{\{n_x n_y n_z\}}(\mathbf{r}) = \Psi_{n_x}(x)\Psi_{n_y}(y)\Psi_{n_z}(z) \;,\quad \Psi_{n_x}(x) = \frac{1}{\sqrt{2^{n_x}\,n_x!}}\left(\frac{\mu\omega}{\pi\hbar}\right)^{1/4} e^{-\frac{\mu\omega x^2}{2\hbar}}\, H_{n_x}\left(\sqrt{\frac{\mu\omega}{\hbar}}x\right) \;,$$

where $H_n(z) = (-1)^n e^{z^2}\frac{d^n}{dz^n}e^{-z^2}$ are the Hermite polynomials.

In order to describe electromagnetic interactions of a QDO with other species or external fields, the drudon acquires a negative charge $(-q)$ opposite to the charge q of the related pseudo-nucleus possessing an infinite mass. The introduction of the electric charges completes the QDO parameter set to $\{q, \mu, \omega\}$, making it different to the quantum harmonic oscillator (possessing just μ and ω).

Within the QDO model[1] one obtains the following multipole polarizabilities

$$\alpha_l = \left(\frac{q^2}{\mu\omega^2}\right)\left[\frac{(2l-1)!!}{l}\right]\left(\frac{\hbar}{2\mu\omega}\right)^{l-1} \;:\;\; \alpha_1 = \frac{q^2}{\mu\omega^2} \;,\quad \alpha_2 = \frac{3\hbar}{4\mu\omega}\alpha_1 \;,\quad \alpha_3 = \frac{5\hbar^2}{4(\mu\omega)^2}\alpha_1 \;,\quad ...$$

and the first three dispersion coefficients (solely expressed in terms of the QDO parameters)

$$C_6 = \frac{3}{4}\frac{\hbar\, q^4}{\mu^2\omega^3} \;,\quad C_8 = \frac{5\hbar}{\mu\omega}\,C_6 \;,\quad C_{10} = \frac{245\hbar^2}{8(\mu\omega)^2}\,C_6 \;,$$

delivering the leading-order contributions to the dispersion energy, $E_{\text{disp}} = -\sum\limits_{n=3,4,...} C_{2n}/R^{2n}$.

In the presence of an external (uniform) electric field $\mathbf{E}$, the QDO Hamiltonian modifies to

$$\hat{\mathcal{H}} = -\frac{\hbar^2}{2\mu}\nabla_{\mathbf{r}}^2 + \frac{1}{2}\mu\omega^2\mathbf{r}^2 - q\left(\mathbf{r}\cdot\mathbf{E}\right) ,$$

which can be straightforwardly diagonalized by means of the coordinate transformation used in Ref. 2: $\mathbf{r} = \tilde{\mathbf{r}} + q\mathbf{E}/\mu\omega^2$. Consequently, the ground-state density acquires shifted coordinate

$$\rho(\mathbf{r}) = \rho_0(\mathbf{r}) = \frac{1}{(\sqrt{2\pi}\sigma)^3}e^{-\frac{\mathbf{r}^2}{4\sigma^2}} , \quad \rho_E(\mathbf{r}) = \rho_0(\tilde{\mathbf{r}}) = \frac{1}{(\sqrt{2\pi}\sigma)^3}e^{-\frac{(\mathbf{r}-\alpha_1\mathbf{E}/q)^2}{4\sigma^2}} , \quad \sigma = \sqrt{\frac{\hbar}{2\mu\omega}} .$$

Substituting the above densities into Eq. (7) of the main manuscript leads there to Eq. (8).

Polarization potentials in different electric field strengths

In Fig. 1 of the main manuscript as well as Figs. S 2 and S 3 below, we present polarization potentials of different atoms calculated within the quantum Drude oscillator (QDO) model in comparison to the results computed by means of density-functional theory (DFT). The polarization potential is defined by Eq. (7) of the main manuscript as the difference of the electrostatic potential of the atoms under an external electric field of different strengths. To obtain this function, in accordance with the usual procedure in molecular physics, only the electronic charge density was used. In principle, the polarization potential could be calculated using free atoms as reference. However, the symmetry of a free atom is different from the one of systems possessing a specified direction defined by the electric field, which leads to different occupation numbers in these two cases. Since these effects are out of the scope of the current study, we took the difference between two non-zero field strengths to ensure that the two systems have the same symmetry.

For electronic structure calculations, we employed DFT using the PBE0 functional[3,4] with the aug-cc-pVQZ basis set and def2-ECP effective core potentials, as implemented in version 5.4 of the Q-Chem code.[5] A DIIS convergence threshold of 10^{-9} a.u. was set to ensure

tight convergence of the electronic density. The density is then exported in Molden format to a formatted checkpoint file, from which the electronic part of the electrostatic potential is calculated using the implementation of Eq. (7) in version 3.8 of Multiwfn.[6,7]

The plots in Fig. 1 of the main manuscript were calculated by considering the field strengths needed to form dipoles of magnitudes 0.01 a.u. and 0.001 a.u. based on reference polarizability values.[8,9] It should be noted that this does not imply that the magnitude of the dipole moment formed was equal to this value as the computation is only approximate. In order to verify that the perceived agreement between the QDO model and the DFT results is not restricted to these field values, we have calculated the polarization potential for Na, P and Ar for different orders of magnitudes of field strengths. The reference field strength was 10^{-6} a.u. for Na and 10^{-5} a.u. for P and Ar. The results are shown in Fig. S 1 below. We have found that the OQDO and DFT curves qualitatively agree within these limits, proving that the values used in the main manuscript fall well within the linear response approximation assumed in the QDO model.

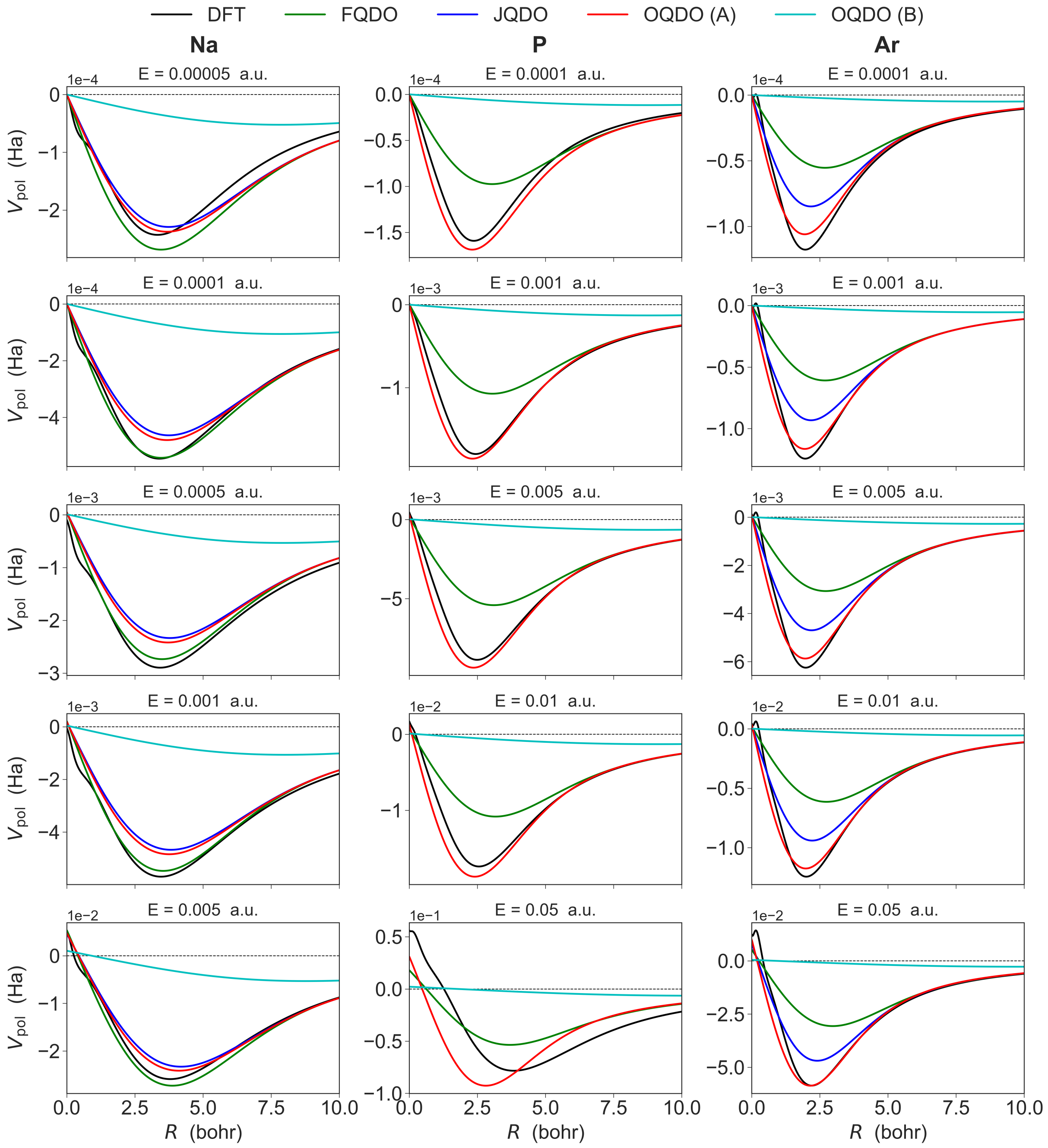

FIG. S1: Polarization potentials for Na, P and Ar (left to right) corresponding to various field strengths (top to bottom) calculated with DFT-PBE0 and various QDO parametrizations. The reference electric field values are 10^{-6} for Na and 10^{-5} a.u. for P and Ar. In all cases the direction along the applied field was chosen for the plots.

Polarization potentials of different elements

To demonstrate that the success of the OQDO model in reproducing the polarization potential is not limited to four elements, below we show the polarization potentials $V_{\mathrm{pol}}(\mathbf{r})$ for 21 elements from the periodic table calculated using the same approach as for Fig. 1 of the main manuscript.

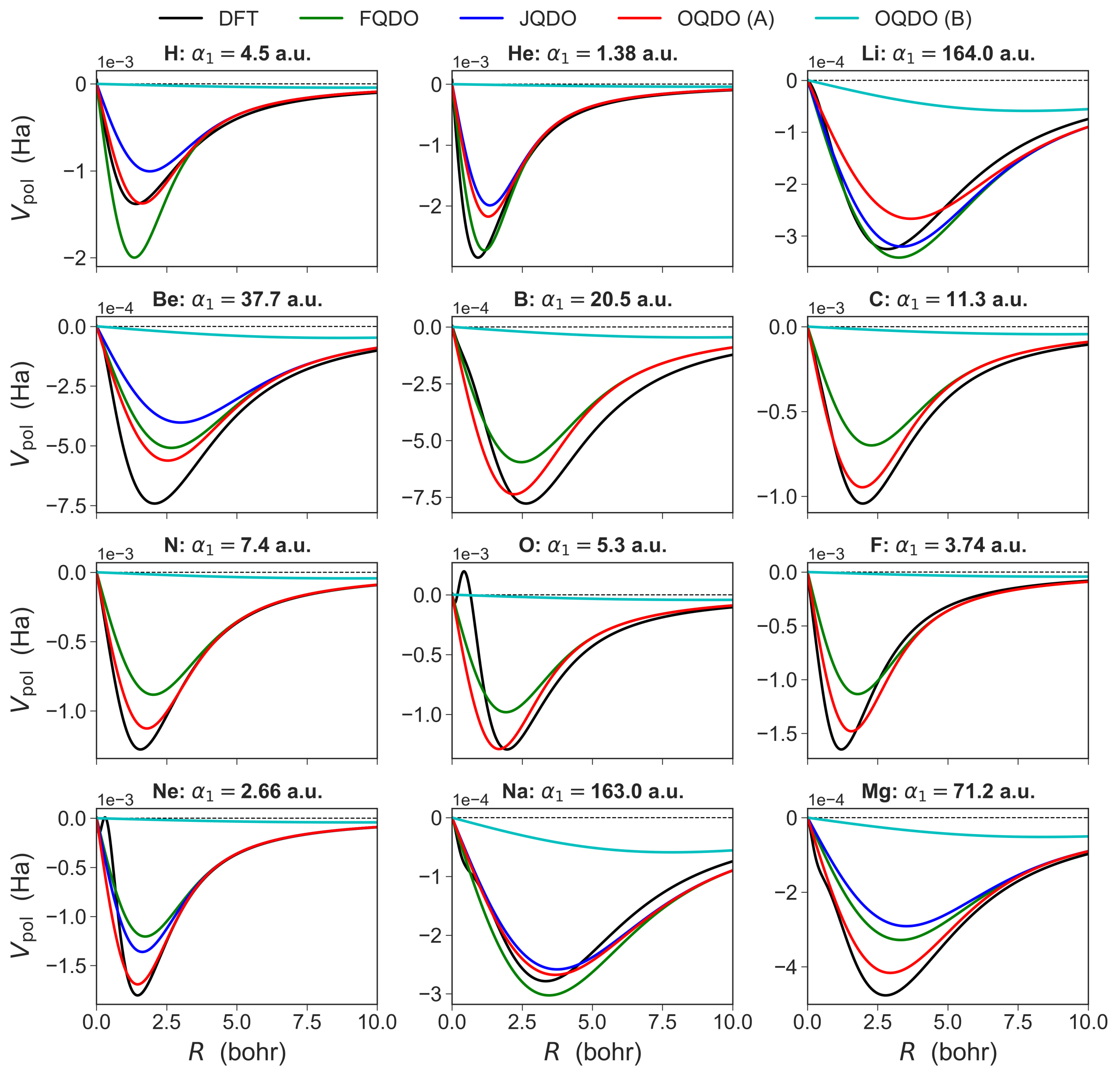

FIG. S 2: Polarization potentials for the first 15 elements (H – P) calculated with DFT-PBE0 and various QDO parametrizations. In all cases the direction along the applied field was chosen for the plots. The reference values for dipole polarizability α_1 are shown for each element. Note that the JQDO curves are shown only when the accurate values of C_8 disperison coefficient are available.[10–12]

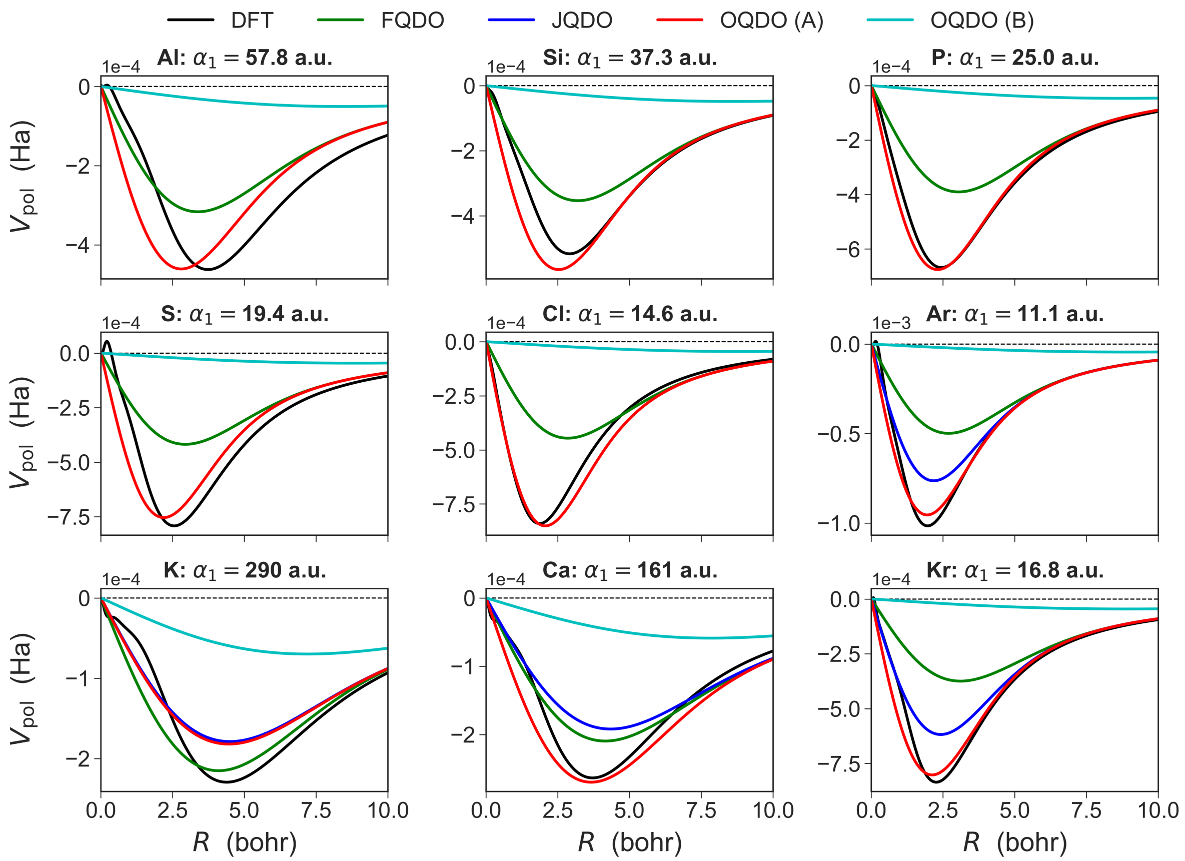

FIG. S 3: Polarization potentials for the 9 elements (Al – Ca and Kr) calculated with DFT-PBE0 and various QDO parametrizations. In all cases the direction along the applied field was chosen for the plots. The reference values for dipole polarizability α_1 are shown for each element. Note that the JQDO curves are shown only when the accurate values of C_8 disperison coefficient are available.[10–12]

Comparison between PBE0 and MP2/CCSD(T) calculations

To demonstrate that the agreement between the OQDO and DFT polarization potential is not due to our choice of the PBE0 functional, we have also calculated this quantity using MP2 and CCSD(T). *Ab initio* curves shown in Fig. S 4 were calculated using an orbital-optimization step from converged wave functions, as implemented in ORCA[13–15] for both MP2 and CCSD(T) projected densities. The basis set used for the calculation was aug-cc-pVQZ (uncontracted for the CCSD(T) curves), except for Si/MP2, where aug-cc-pVTZ was employed. As shown by Fig. S 4, our PBE0 results are in good agreement with the performed MP2 and CCSD(T) calculations.

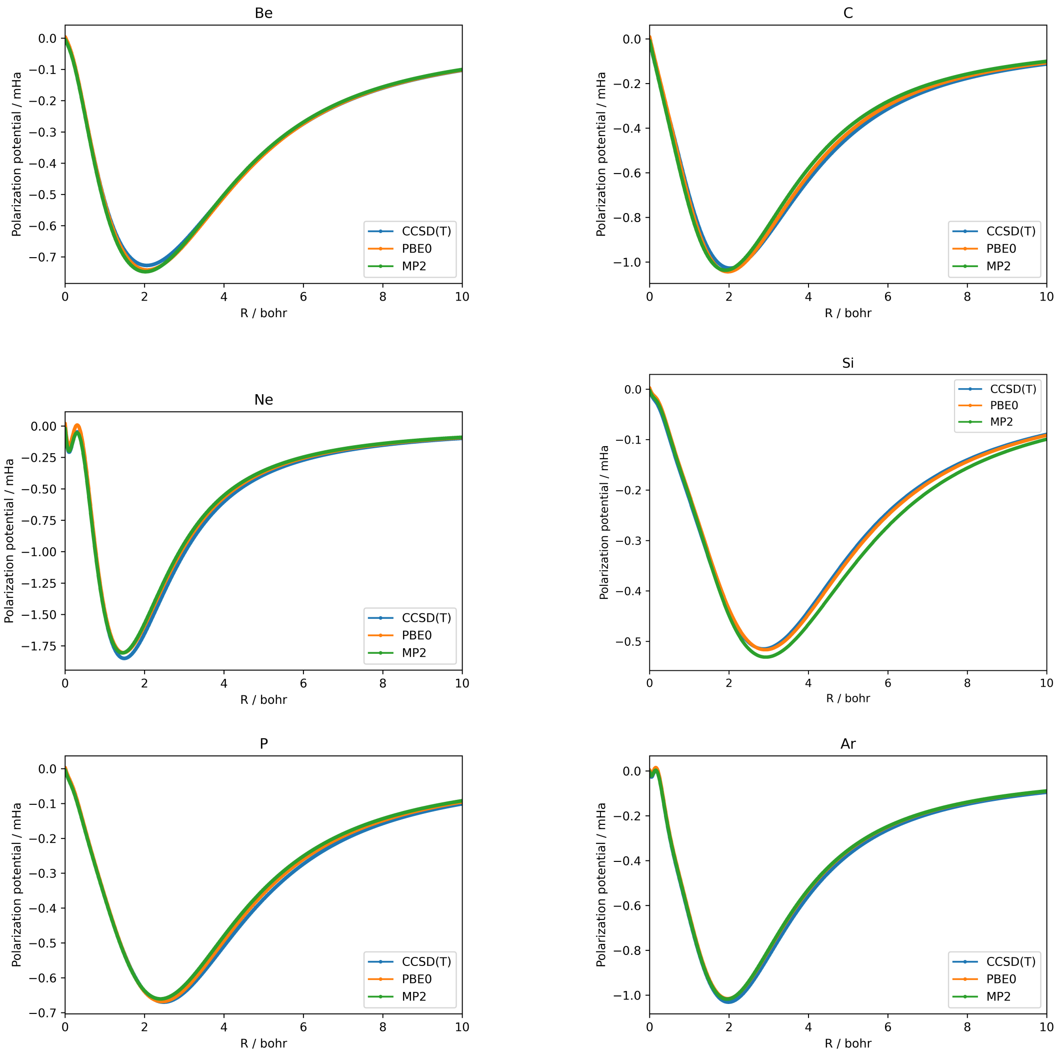

FIG. S 4: Comparison between PBE0 and orbital-optimized MP2/CCSD(T) calculations of the polarization potential, given by Eq. (7) of the main manuscript, performed for Be, C, Ne, Si, P, and Ar atoms.

Optimized Quantum Drude Oscillator (OQDO) parameters for the periodic table

One of the main advantages of our proposed OQDO scheme is that oscillators can be parametrized for most free atoms since both α_1 and C_6 values are available for them. The values used, also shown in Table 1, with the exception of Cs, where the values suggested by Derevianko *et al.*[16] were used, are taken from Ref. 8. As explained in the main text, our equations have two solutions, A and B. In Table 2, we present the three OQDO parameters

corresponding to both solutions. Note that the OQDO model is defined using solution A, whereas the second solution (B) is solely shown for comprehensiveness. The polarizability values presented here may be slightly different from the ones shown in Figs. S 2, and S 3 for some cases, since the values used for creating the figures were taken from Ref. 9.

TABLE S 1: Reference values of dipole polarizability and dipole-dipole dispersion coefficients (in atomic units)

Atom	α_1	C_6	Atom	α_1	C_6	Atom	α_1	C_6	Atom	α_1	C_6
H	4.5	6.5	He	1.38	1.46	Li	164.2	1387.0	Be	38.0	214.0
B	21.0	99.5	C	12.0	46.6	N	7.4	24.2	O	5.4	15.6
F	3.8	9.52	Ne	2.67	6.38	Na	162.7	1556.0	Mg	71.0	627.0
Al	60.0	528.0	Si	37.0	305.0	P	25.0	185.0	S	19.6	134.0
Cl	15.0	94.6	Ar	11.1	64.3	K	292.9	3897.0	Ca	160.0	2221.0
Sc	120.0	1383.0	Ti	98.0	1044.0	V	84.0	832.0	Cr	78.0	602.0
Mn	63.0	552.0	Fe	56.0	482.0	Co	50.0	408.0	Ni	48.0	373.0
Cu	42.0	253.0	Zn	40.0	284.0	Ga	60.0	498.0	Ge	41.0	354.0
As	29.0	246.0	Se	25.0	210.0	Br	20.0	162.0	Kr	16.8	129.6
Rb	319.2	4691.0	Sr	199.0	3170.0	Y	126.74	1968.58	Zr	119.97	1677.91
Nb	101.6	1263.61	Mo	88.42	1028.73	Tc	80.08	1390.87	Ru	65.89	609.75
Rh	56.1	469.0	Pd	23.68	157.5	Ag	50.6	339.0	Cd	39.7	452.0
In	70.22	707.05	Sn	55.95	587.42	Sb	43.67	459.32	Te	37.65	396.0
I	35.0	385.0	Xe	27.3	285.9	Cs	399.9	6846.0	Ba	275.0	5727.0
La	213.7	3884.5	Ce	204.7	3708.33	Pr	215.8	3911.84	Nd	208.4	3908.75
Pm	200.2	3847.68	Sm	192.1	3708.69	Eu	184.2	3511.71	Gd	158.3	2781.53
Tb	169.5	3124.41	Dy	164.64	2984.29	Ho	156.3	2839.95	Er	150.2	2724.12
Tm	144.3	2576.78	Yb	138.9	2387.53	Lu	137.2	2371.8	Hf	99.52	1274.8
Ta	82.53	1019.92	W	71.04	847.93	Re	63.04	710.2	Os	55.06	596.67
Ir	42.51	359.1	Pt	39.68	347.1	Au	36.5	298.0	Hg	33.9	392.0
Tl	69.92	717.44	Pb	61.8	697.0	Bi	49.02	571.0	Po	45.01	530.92
At	38.93	457.53	Rn	33.54	390.63	Fr	317.8	4224.44	Ra	246.2	4851.32
Ac	203.3	3604.41	Th	217.0	4047.54	Pa	154.4	2367.42	U	127.8	1877.1
Np	150.5	2507.88	Pu	132.2	2117.27	Am	131.2	2110.98	Cm	143.6	2403.22
Bk	125.3	1985.82	Cf	121.5	1891.92	Es	117.5	1851.1	Fm	113.4	1787.07
Md	109.4	1701.0	No	105.4	1578.18						

TABLE S2: Parameters for OQDO model for 102 elements from the periodic table computed using the reference values from Ref. 8. All the parameters are presented in atomic units.

Atom	q_A	ω_A	μ_A	q_B	ω_B	μ_B	Atom	q_A	ω_A	μ_A	q_B	ω_B	μ_B
H	0.8295	0.428	0.8348	0.1466	0.428	0.0261	He	0.8927	1.0222	0.5527	0.1233	1.0222	0.0105
Li	0.8828	0.0686	1.0088	0.4153	0.0686	0.2233	Be	1.045	0.1976	0.736	0.3073	0.1976	0.0637
B	1.0933	0.3008	0.6289	0.2757	0.3008	0.04	C	1.1146	0.4315	0.5561	0.2455	0.4315	0.027
N	1.1297	0.5892	0.4967	0.2227	0.5892	0.0193	O	1.1313	0.7133	0.4658	0.208	0.7133	0.0157
F	1.1296	0.879	0.4345	0.1925	0.879	0.0126	Ne	1.182	1.1933	0.3675	0.1869	1.1933	0.0092
Na	0.9419	0.0784	0.8877	0.4415	0.0784	0.195	Mg	1.1289	0.1658	0.6526	0.3972	0.1658	0.0808
Al	1.174	0.1956	0.6007	0.3928	0.1956	0.0672	Si	1.272	0.2971	0.4956	0.3714	0.2971	0.0422
P	1.3154	0.3947	0.4443	0.3466	0.3947	0.0308	S	1.333	0.4651	0.4191	0.3304	0.4651	0.0257
Cl	1.3554	0.5606	0.3897	0.3148	0.5606	0.021	Ar	1.3835	0.6958	0.3562	0.2992	0.6958	0.0167
K	0.9114	0.0606	0.7731	0.5681	0.0606	0.3003	Ca	1.1403	0.1157	0.6074	0.531	0.1157	0.1317
Sc	1.1266	0.1281	0.645	0.471	0.1281	0.1127	Ti	1.143	0.1449	0.6345	0.4457	0.1449	0.0965
V	1.1465	0.1572	0.6331	0.4252	0.1572	0.0871	Cr	1.0311	0.1319	0.783	0.3735	0.1319	0.1028
Mn	1.1577	0.1854	0.6187	0.393	0.1854	0.0713	Fe	1.1804	0.2049	0.5924	0.387	0.2049	0.0637
Co	1.1805	0.2176	0.5887	0.3746	0.2176	0.0593	Ni	1.1631	0.2159	0.6049	0.3649	0.2159	0.0595
Cu	1.0563	0.1912	0.7264	0.3192	0.1912	0.0664	Zn	1.1597	0.2367	0.6003	0.3458	0.2367	0.0534
Ga	1.1402	0.1844	0.6369	0.3814	0.1844	0.0713	Ge	1.2716	0.2808	0.5002	0.3818	0.2808	0.0451
As	1.3629	0.39	0.4211	0.3731	0.39	0.0316	Se	1.4014	0.448	0.3914	0.3692	0.448	0.0272
Br	1.4446	0.54	0.3578	0.3599	0.54	0.0222	Kr	1.4635	0.6122	0.3401	0.3493	0.6122	0.0194
Rb	0.9236	0.0614	0.7092	0.6091	0.0614	0.3085	Sr	1.1431	0.1067	0.5764	0.5833	0.1067	0.1501
Y	1.2886	0.1634	0.4906	0.5493	0.1634	0.0892	Zr	1.2412	0.1554	0.5315	0.5188	0.1554	0.0929
Nb	1.2234	0.1632	0.553	0.4828	0.1632	0.0861	Mo	1.2265	0.1754	0.5527	0.4624	0.1754	0.0786
Tc	1.5366	0.2892	0.3526	0.5613	0.2892	0.047	Ru	1.1769	0.1873	0.5995	0.4049	0.1873	0.0709
Rh	1.1628	0.1987	0.6105	0.3814	0.1987	0.0657	Pd	1.2619	0.3745	0.4795	0.3279	0.3745	0.0324
Ag	1.0667	0.1765	0.7215	0.3397	0.1765	0.0732	Cd	1.4711	0.3824	0.3728	0.4378	0.3824	0.033
In	1.2087	0.1912	0.5692	0.4239	0.1912	0.07	Sn	1.3039	0.2502	0.4854	0.4274	0.2502	0.0522
Sb	1.3832	0.3211	0.4249	0.4226	0.3211	0.0397	Te	1.4311	0.3725	0.3921	0.4198	0.3725	0.0337
I	1.488	0.419	0.3603	0.428	0.419	0.0298	Xe	1.5348	0.5115	0.3298	0.4136	0.5115	0.024
Cs	0.8789	0.0571	0.5929	0.7144	0.0571	0.3917	Ba	1.1688	0.101	0.4873	0.7016	0.101	0.1756
La	1.1934	0.1134	0.5181	0.6291	0.1134	0.144	Ce	1.2081	0.118	0.5121	0.6244	0.118	0.1368
Pr	1.1879	0.112	0.5213	0.6291	0.112	0.1462	Nd	1.2222	0.12	0.4977	0.6368	0.12	0.1351
Pm	1.2532	0.128	0.4788	0.6413	0.128	0.1254	Sm	1.2725	0.134	0.4694	0.6393	0.134	0.1185
Eu	1.2811	0.138	0.4679	0.6321	0.138	0.1139	Gd	1.287	0.148	0.4777	0.5967	0.148	0.1027
Tb	1.2918	0.145	0.4683	0.6156	0.145	0.1064	Dy	1.2922	0.1468	0.4706	0.6086	0.1468	0.1044
Ho	1.3136	0.155	0.4595	0.606	0.155	0.0978	Er	1.3276	0.161	0.4527	0.603	0.161	0.0934
Tm	1.3325	0.165	0.4519	0.596	0.165	0.0904	Yb	1.3215	0.165	0.4618	0.5826	0.165	0.0898
Lu	1.3298	0.168	0.4567	0.5836	0.168	0.088	Hf	1.2483	0.1716	0.5316	0.4893	0.1716	0.0817
Ta	1.2863	0.1997	0.503	0.4744	0.1997	0.0684	W	1.3123	0.224	0.483	0.4618	0.224	0.0598
Re	1.3126	0.2383	0.4814	0.4456	0.2383	0.0555	Os	1.3298	0.2624	0.4664	0.4339	0.2624	0.0496
Ir	1.2474	0.265	0.5214	0.3783	0.265	0.0479	Pt	1.2896	0.2939	0.4851	0.3837	0.2939	0.043
Au	1.2698	0.2982	0.4966	0.3694	0.2982	0.042	Hg	1.5367	0.4548	0.3367	0.4383	0.4548	0.0274
Tl	1.2215	0.1957	0.5573	0.4278	0.1957	0.0684	Pb	1.3196	0.2433	0.4759	0.4454	0.2433	0.0542
Bi	1.417	0.3168	0.4081	0.4472	0.3168	0.0406	Po	1.4546	0.3494	0.385	0.4482	0.3494	0.0365
At	1.5013	0.4025	0.3573	0.4444	0.4025	0.0313	Rn	1.5459	0.463	0.3324	0.4396	0.463	0.0269
Fr	0.8801	0.0558	0.7836	0.5787	0.0558	0.3388	Ra	1.1841	0.1067	0.5001	0.6692	0.1067	0.1597
Ac	1.1978	0.1163	0.5219	0.6171	0.1163	0.1386	Th	1.2028	0.1146	0.5076	0.6386	0.1146	0.1431
Pa	1.211	0.1324	0.5417	0.556	0.1324	0.1142	U	1.2502	0.1532	0.5208	0.5345	0.1532	0.0952
Np	1.2718	0.1476	0.4931	0.5781	0.1476	0.1019	Pu	1.2933	0.1615	0.4849	0.5599	0.1615	0.0909
Am	1.299	0.1635	0.4811	0.5608	0.1635	0.0896	Cm	1.2917	0.1554	0.4812	0.5767	0.1554	0.0959
Bk	1.3057	0.1686	0.4784	0.5543	0.1686	0.0862	Cf	1.3052	0.1709	0.4801	0.548	0.1709	0.0846
Es	1.3247	0.1788	0.4673	0.5497	0.1788	0.0805	Fm	1.3377	0.1853	0.4596	0.5482	0.1853	0.0772
Md	1.3415	0.1895	0.4581	0.5429	0.1895	0.075	No	1.3295	0.1894	0.4674	0.5313	0.1894	0.0746

Two solutions within the Tang-Toennies model

As it was discussed in (the Supplemental Material of) Ref. 17, the scaling law $R_{\text{vdW}} \propto \alpha_1^{1/7}$ can be also derived from the Tang-Toennies (TT) model,[18] which is known to accurately describe the binding curves of noble gas dimers. Within the TT model with the exchange energy in the Born-Mayer form, $E_{\text{ex}} = Ae^{-bR}$, the force balance with the dipolar van der Waals dispersion attraction delivers the following formula

$$\alpha_1 = \frac{2^7 \cdot \alpha_1 \cdot Ab \cdot R_{\text{vdW}}^7}{6C_6 \cdot \exp(2bR_{\text{vdW}})} \;, \tag{S 1}$$

which we compare to Eq. (9) of the main manuscript. Although the proportionality function here formally depends on α_1, due to $C_6 \propto \alpha_1^2$ and the direct proportionality of the exchange repulsive energy to the dipole polarizability,[19] this dependence disappears.[17] Therefore, in analogy to the QDO case, we can impose a condition that parameter b should deliver the same R_{vdW} as that obtained from Eq. (4). This allows us to derive the following equation analogous to Eq. (10)

$$x = k\, e^{m\, x} \quad , \quad x = b \cdot a_0 \;, \quad k = \frac{6C_6 \cdot (4\pi\epsilon_0) \cdot \alpha_{\text{fsc}}^{4/3}}{2^7 \cdot \alpha_1 A \cdot a_0^3} \;, \quad m = \frac{2R_{\text{vdW}}}{a_0} = \frac{2(\alpha_1/4\pi\varepsilon_0)^{1/7}}{\alpha_{\text{fsc}}^{4/21}\, a_0^{3/7}} \;, \tag{S 2}$$

where we introduced the dimensionless coefficients k and m as well as used Eq. (4) to express R_{vdW} in terms of α_1. Similar to Eq. (10), this transcendental equation also has two solutions, as illustrated by Fig. S 5 using the example of Ar with the TT parameters $A = 748.3$ a.u., $C_6 = 64.3$ a.u. from Ref. 18 and $\alpha_1 = 11.1$ a.u. from Ref. 8. The first solution gives $b_{\text{A}} = 1.756$ a.u., which is comparable to the actual TT parameter $b = 2.031$ a.u.,[18] whereas the second solution leads to very small value $b_{\text{B}} = 5.7 \times 10^{-6}$ a.u. Thus, the existence of two solutions does not depend on the specific model (depending on the choice of the exchange energy) for vdW-bonded dimers.

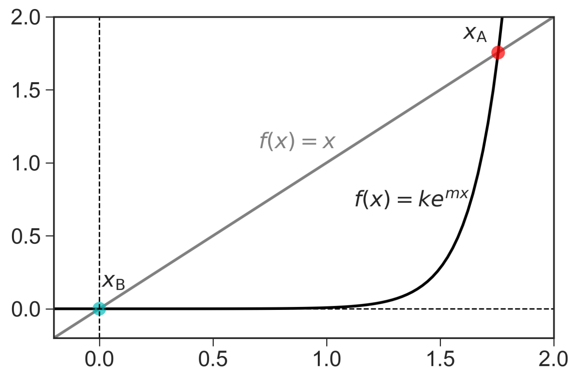

FIG. S 5: Two solutions (for $x = b \cdot a_0$) of the transcendental equation given by Eq. (S 2) for argon.

Reproducing multipole polarizabilities α_2 and α_3

In addition to our discussion of the dispersion coefficients C_8 and C_{10} performed in the main text, here we compare the quadrupole (α_2) and octupole (α_3) polarizabilities obtained by means of the FQDO, JQDO, and OQDO models. For the latter, we also show the polarizabilities obtained by using the simple rescaling $\alpha_2 \to \sqrt{2}\,\alpha_2$ and $\alpha_3 \to 2\,\alpha_3$. These correction factors are based on multipole polarizabilities of hydrogen-like atoms with a varying ratio of electron-to-proton mass. This rescaling makes the OQDO results comparable to the JQDO model. Among the four considered schemes, the scaled OQDO delivers the most accurate α_2 and α_3 for noble-gas atoms, which are known to be the best test systems for the QDO model (as discussed in Ref. 6 of the main manuscript). Thus, similar to C_8 and C_{10}, the accuracy for α_2 and α_3 can be significantly improved by the rescaling factors, the same for all species.

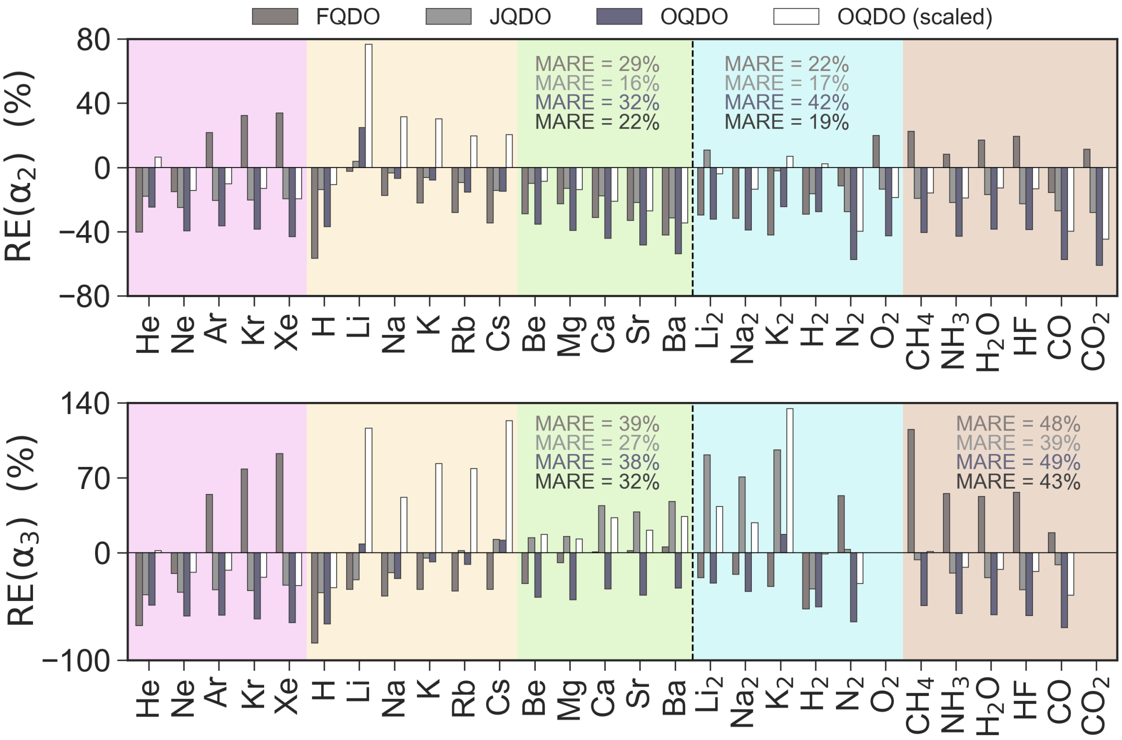

FIG. S 6: Multipole polarizabilities α_2 and α_3 obtained by the FQDO, JQDO, and OQDO models. Scaled OQDO means using the aforementioned corrected formulas: $\alpha_2 \to \sqrt{2}\,\alpha_2$ and $\alpha_3 \to 2\,\alpha_3$. Relative error RE $= (\alpha_j - \alpha_j^{\text{ref}})/\alpha_j^{\text{ref}}$ with respect to *ab initio* reference data[10–12,20] is plotted. Numerical values of mean absolute relative errors (MARE) evaluated separately for atoms and molecules are displayed for the four computational schemes. For O_2 and CO_2, no reliable *ab initio* reference data for α_3 was found.

Reproducing C_8 and C_{10} dispersion coefficients

In addition to our discussion of the dispersion coefficients C_8 and C_{10} performed in the main manuscript, here we extend the analysis to include also JQDO and scaled OQDO models. The latter correspond to the simple rescaling of OQDO dispersion coefficients as $C_8 \to 1.3 \times C_8^{\text{OQDO}}$ and $C_{10} \to 1.5 \times C_{10}^{\text{OQDO}}$. These correction factors were adjusted to minimize the error across all the systems considered. Within the JQDO model, C_8 dispersion coefficients are fixed to their reference values, therefore the errors shown are zero. However, C_{10}^{JQDO} is not exact and calculated via the QDO relations (given within the first section of this Supplemental Material).

Overall, the rescaling of OQDO dispersion coefficients significantly improves the statisti-

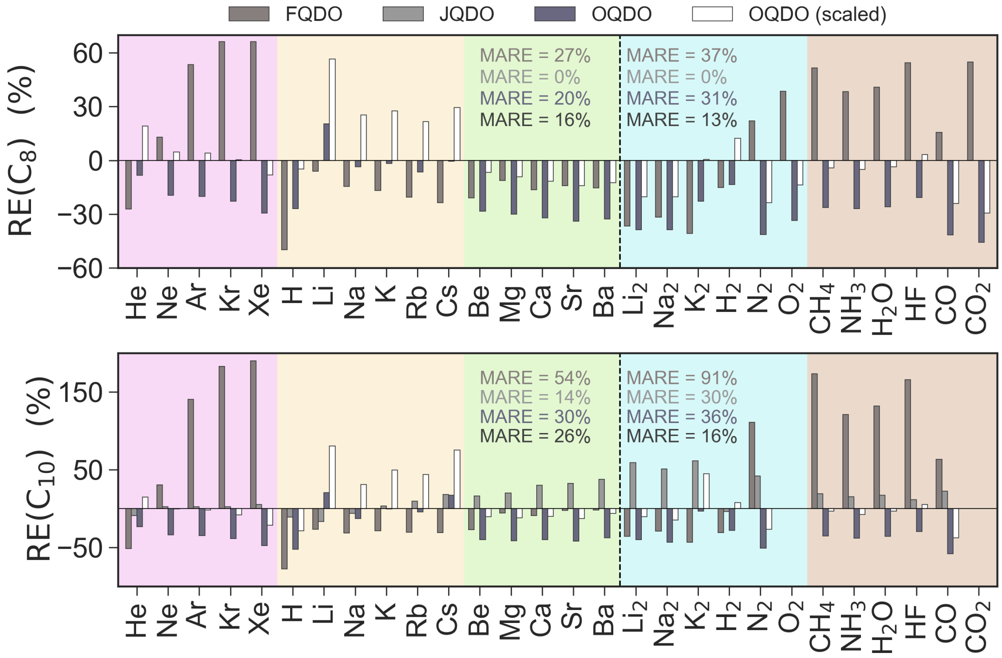

FIG. S 7: Extended version of Fig. 3 in the main manuscript with C_8 and C_{10} dispersion coefficients obtained by the FQDO, JQDO, OQDO, and scaled OQDO models, in analogy to Fig. S 6.

cal accuracy of the model, although for alkali metals scaled OQDO is less accurate than the original OQDO. For C_8, scaled OQDO is the most accurate model among the ones considered (besides JQDO, where C_8 is exact). For C_{10}, JQDO has smaller error for atoms than scaled OQDO, but for molecules scaled OQDO (16 %) beats JQDO (30 %). When computing the total statistics for C_{10}, scaled OQDO and JQDO have almost the same MARE of 21.7 % and 20.9 %, respectively.

The scaling could also be done for FQDO and JQDO models (the latter for C_{10} only.) For FQDO we found the optimal scaling coefficients to be 0.82 and 0.45 for C_8 and C_{10}, respectively, whereas for JQDO we found the scaling coefficient to be 0.86. Table S 3 summarizes the statistical analysis of errors for both original and rescaled QDO models. As one can see, the scaling of FQDO also lowers the errors, however, their magnitude is still large being 29.1% for C_8 and 49.1% for C_{10}. Thus, the uniformly scaled OQDO model is still twice more accurate than the uniformly scaled FQDO model.

We emphasize that the direct comparison of OQDO to JQDO in terms of dispersion

coefficients is not fully fair, since JQDO is by construction is better suited to describe dispersion coefficients due to C_8 being fixed to its exact value. Nevertheless, for the sake of completeness, in Table S 3 we also show our analysis for the scaled JQDO model. Not surprisingly, this scaling allows to achieve the smallest errors across all the QDO models considered. Since the requirement to know accurate C_8 coefficients prevents the JQDO model to be generalized well beyond the considered set of 28 systems, we conclude that the presented OQDO model delivers the most efficient QDO approach with respect to both the feasibility and accuracy. We also note that an error of 20% in the C_{10} coefficient would translate to an error of 2% in the overall dispersion energy for an atomic dimer, given that the C_{10} term in the dispersion energy contributes roughly 10% to the multipole expansion of the dispersion energy given by $E_{\text{disp}} = -\sum\limits_{n=3,4,...} C_{2n}/R^{2n}$.

TABLE S 3: MARE of the QDO models considered for C_8 and C_{10} dispersion coefficients calculated on the set of 16 atoms and 12 small molecules. The scaled values correspond to the uniform scaling of dispersion coefficients, where the scaling coefficients are given in the above text.

	MARE in C_8, %			MARE in C_{10}, %		
Model	atoms	molecules	all	atoms	molecules	all
FQDO	27.2	36.7	**31.3**	54.3	90.7	**68.3**
Scaled FQDO	32.2	25.0	**29.1**	57.1	36.2	**49.1**
OQDO	19.8	31.3	**24.7**	30.5	36.5	**32.8**
Scaled OQDO	16.0	13.4	**14.9**	25.5	16.2	**21.9**
JQDO	–	–	–	13.8	30.2	**20.1**
Scaled JQDO	–	–	–	12.9	15.5	**13.9**

Furthermore, the main advantage of the presented OQDO model is its accurate description of the polarization potential, which is crucial to build advanced force fields based on coupled QDOs, to properly capture molecular interactions in complex systems at both short and long ranges.

Calculations of atomic multipolar polarizabilities with finite field approach

This section presents a short discussion related to numerical evaluations of multipole polarizabilities, illustrating main challenges through a few representative cases. Computing higher-order polarizabilities using standard electronic-structure codes is a substantial challenge for both atoms and molecules. While accurate methods such as coupled-perturbed Hartree-Fock and density-functional perturbation theory are available for dipolar response, they are not implemented for quadrupolar and higher-order response. One must therefore resort to finite field approaches, if available in an electronic-structure code. Finite field calculations, however, also suffer from serious technical difficulties, general for all quantum-chemistry methods:

- It is crucial to carefully choose a strength of an applied electric field, striking a balance between having it being perturbatively small yet large enough for numerical purposes. Higher multipole polarizabilities possess larger magnitudes requiring weaker electric fields. Thus, calculations of α_l become more and more problematic with the increase of the order l.

- Open shell atoms in external fields present an additional drawback because of the competing electronic states. Let us consider the O atom which has $2s^2 2p^4$ configuration of the valence shell corresponding to the three degenerate states (see Fig. S 8). A corresponding free atom possesses spherically symmetric charge density, which is a superposition of the three states. Applying external fields breaks the spherical symmetry making the situation difficult to handle numerically, since a wave function (as a linear combination of different orbitals) does not remain fixed between calculations for different field strengths (and even during the self-consistent procedure). To tackle this problem, the occupancy of each orbital needs to be explicitly controlled (for instance, based on the irreducible representations of the point group related to a considered system). However, such a precise control is normally not available in widely used

electronic-structure codes.

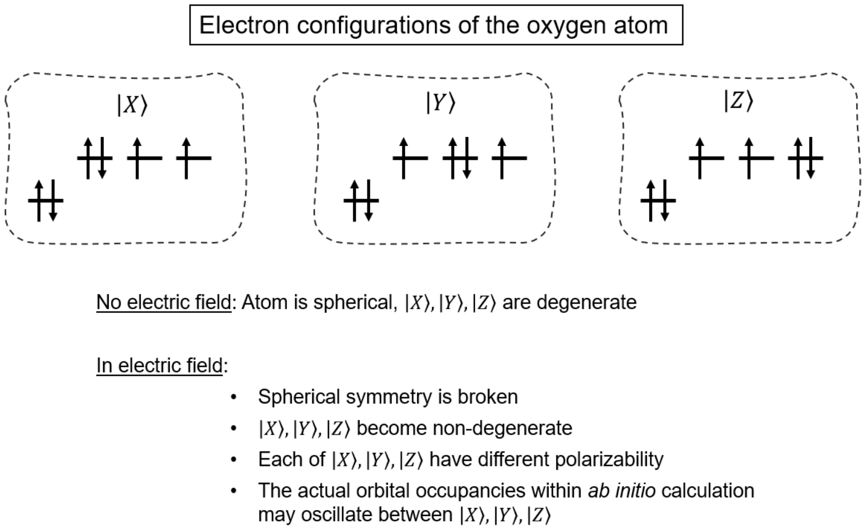

FIG. S 8: Schematic representation of the difficulties with open-shell systems.

- In addition, any intention to obtain accurate higher-order multipole polarizabilities faces a serious problem regarding basis sets. Highly diffuse basis functions up to order h ($l = 5$) are already required for dipole polarizabilities, and even larger angular momenta are needed for higher-order polarizabilities. To our knowledge, there are no ready-to-use optimized basis sets with such high angular momenta included in standard quantum-chemical codes.

Altogether, this makes numerical evaluations of the multipole polarizabilities more and more demanding with the increase of l, as can be already seen for the quadrupole polarizability (α_2). The fact that there are no reference values of α_2 in literature present for many atoms, although there are even a few different databases of α_1 almost for the entire periodic table,[8,9,21] supports the above statement. This fact makes our OQDO model valuable, as delivering valuable estimates of α_2, α_3, C_8, and C_{10} solely from the well tabulated dipolar response quantities (α_1 and C_6).

To further support our statements, we additionally performed a number of finite-field calculations of α_1 and α_2. Perturbations by external electric fields were added to the core

TABLE S 4: Comparison of our PBE0 and CCSD(T) results for the dipole and quadrupole polarizabilities. The reference values of α_1 and α_2, if known (otherwise, labeled with "-"), are taken from Ref. 72 in the main manuscript. For the column "CCSD(T)", we have performed HF and CCSD calculations for the H and He atoms, respectively. There are no JQDO values for N and P atoms, since there is no accurate C_8 coefficient (required to perform the JQDO parametrization) in literature for those atoms.

Atom	$\alpha_{1;aVQZ}^{\text{CCSD(T)}}$	$\alpha_{1;aVQZ}^{\text{PBE0}}$	α_1^{OQDO}	α_1^{JQDO}	Ref.	$\alpha_{2;aVQZ}^{\text{CCSD(T)}}$	$\alpha_{2;aVQZ}^{\text{PBE0}}$	α_2^{OQDO}	α_2^{JQDO}	Ref.
H	4.49	5.15	4.50	4.50	4.50	10.1	11.9	9.45	12.92	15.00
He	1.38	1.49	1.38	1.38	1.38	1.79	1.99	1.83	2.00	2.44
Li	168.9	147.8	164.0	164.0	164.0	1438.1	1541.6	1776.3	1477.1	1383.0
Be	37.9	43.0	37.7	37.7	37.7	301.0	328.0	193.72	270.33	-
N	7.27	7.75	7.40	-	7.40	24.6	27.9	18.96	-	-
P	25.1	26.5	25.0	-	25.0	168.0	185.0	106.92	-	-
Ne	2.59	2.69	2.66	2.66	2.66	5.03	5.61	4.54	5.64	6.42
Ar	11.1	11.3	11.1	11.1	11.1	41.8	44.2	33.59	41.99	50.21
Kr	16.8	17.2	16.80	16.80	16.80	81.8	85.9	60.51	78.31	95.55

Hamiltonian [an option in the PySCF code: Sun *et al.*, JCP 153, 024109 (2020)] as (in a.u., with $|e| = 1$) $\Delta H = -E_\alpha r_\alpha$ and $\Delta H = 0.5E_{\alpha\beta}(3z^2 - r^2)$, respectively, for the dipole and quadrupole polarizabilities. The polarizabilities were extracted from the numerical derivatives of energy with respect to the strength of applied electric fields (chosen to be 0.001 a.u.). We employed Dunning's (singly augmented) aug-cc-pVXZ basis sets, which are designed to model electronic response properties within the standard electronic-structure codes.

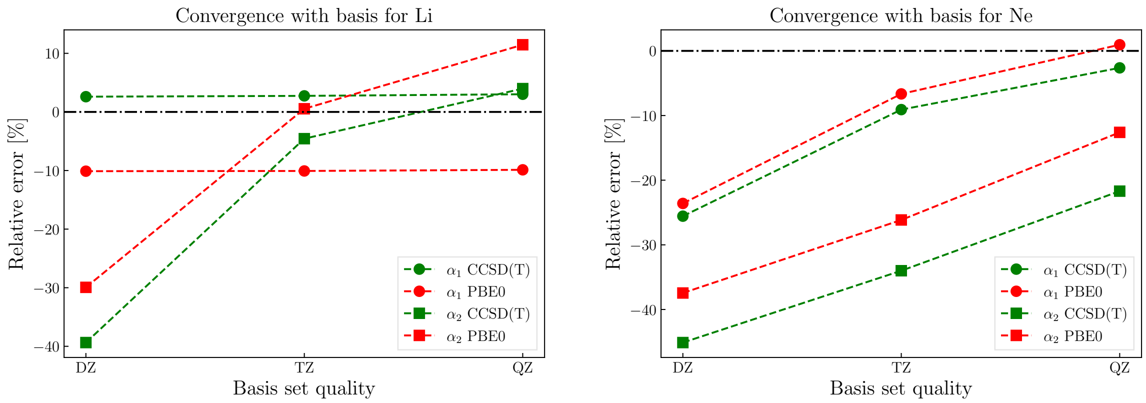

FIG. S 9: Convergence of the CCSD(T) results for the dipole and quadrupole polarizabilities of lithium (left) and neon (right). The basis sets DZ, TZ, and QZ correspond, respectively, to aug-cc-pVDZ, aug-cc-pVTZ, and aug-cc-pVQZ basis sets. The reference values are the same as in Table S 4, taken from Ref. 72 in the main manuscript.

One of the most crucial issues one observes is the slow convergence of the results with the basis set size. Indeed, Fig. S 9 illustrates that even with the aug-cc-pVQZ basis set (quite often the largest basis set implemented in codes) the CCSD(T) results become fully converged neither for α_2 nor for α_1 (and convergence is even slower for PBE0).

To complete our analysis, we performed analogous finite-field calculations for several other atoms. To avoid issues arising from competing electronic states (illustrated by Fig. S 8), here we only present results for atoms possessing symmetric ground states. For Li and Be, the applied field strengths were exceptionally chosen to be 0.0001 a.u. because of their large polarizabilities. Table S 4 summarizes our CCSD(T) and PBE0 results and provides the comparison to the reference values from Ref. 72 of the main manuscript, where the Dunning's basis sets for response quantities were introduced. As one can see, PBE0 consistently overshoots α_1, except for Li where PBE0 value delivers an underestimated dipole polarizability. CCSD(T) with aug-cc-pVQZ basis set shows very good agreement with the reference α_1. For α_2, PBE0 is seemingly closer to the reference data than CCSD(T). However, as was discussed above, such a situation is due to the fact that already for quadrupole polarizabilities it is impossible to converge with the standard basis sets. Remarkably, the difference in the results between the JQDO and OQDO models for the quadrupole polarizability is comparable to the one between the CCSD(T) and PBE0 results.

References

(1) Jones, A. P.; Crain, J.; Sokhan, V. P.; Whitfield, T. W.; Martyna, G. J. Quantum Drude oscillator model of atoms and molecules: Many-body polarization and dispersion interactions for atomistic simulation. *Phys. Rev. B* **2013**, *87*, 144103.

(2) Karimpour, M. R.; Fedorov, D. V.; Tkatchenko, A. Quantum framework for describing retarded and nonretarded molecular interactions in external electric fields. *Phys. Rev. Research* **2022**, *4*, 013011.

(3) Adamo, C.; Barone, V. Toward reliable density functional methods without adjustable parameters: The PBE0 model. *J. Chem. Phys.* **1999**, *110*, 6158–6170.

(4) Perdew, J. P.; Burke, K.; Ernzerhof, M. Generalized Gradient Approximation Made Simple. *Phys. Rev. Lett.* **1996**, *77*, 3865–3868.

(5) Epifanovsky, E., et al. Software for the frontiers of quantum chemistry: An overview of developments in the Q-Chem 5 package. *J. Chem. Phys.* **2021**, *155*, 084801.

(6) Lu, T.; Chen, F. Multiwfn: A multifunctional wavefunction analyzer. *J. Comput. Chem.* **2012**, *33*, 580–592.

(7) Zhang, J.; Lu, T. Efficient evaluation of electrostatic potential with computerized optimized code. *Phys. Chem. Chem. Phys.* **2021**, *23*, 20323–20328.

(8) Gobre, V. V. Efficient modelling of linear electronic polarization in materials using atomic response functions. Ph.D. thesis, Fritz Haber Institute Berlin, 2016.

(9) Schwerdtfeger, P.; Nagle, J. K. 2018 Table of static dipole polarizabilities of the neutral elements in the periodic table. *Mol. Phys.* **2019**, *117*, 1200–1225.

(10) Porsev, S. G.; Derevianko, A. Accurate relativistic many-body calculations of van der Waals coefficients C_8 and C_{10} for alkali-metal dimers. *J. Chem. Phys.* **2003**, *119*, 844–850.

(11) Porsev, S. G.; Derevianko, A. High-accuracy calculations of dipole, quadrupole, and octupole electric dynamic polarizabilities and van der Waals coefficients C_6, C_8, and C_{10} for alkaline-earth dimers. *J. Exp. Theor. Phys.* **2006**, *102*, 195–205.

(12) Jiang, J.; Mitroy, J.; Cheng, Y.; Bromley, M. W. J. Effective oscillator strength distributions of spherically symmetric atoms for calculating polarizabilities and long-range atom–atom interactions. *Atom. Data Nucl. Data* **2015**, *101*, 158–186.

(13) Neese, F. The ORCA program system. *WIREs Computational Molecular Science* **2012**, *2*, 73–78.

(14) Neese, F. Software update: the ORCA program system, version 4.0. *WIREs Computational Molecular Science* **2018**, *8*, e1327.

(15) Veis, L.; Antalík, A.; Brabec, J.; Neese, F.; Legeza, O.; Pittner, J. Coupled Cluster Method with Single and Double Excitations Tailored by Matrix Product State Wave Functions. *J. Phys. Chem. Lett.* **2016**, *7*, 4072–4078.

(16) Derevianko, A.; Porsev, S. G.; Babb, J. F. Electric dipole polarizabilities at imaginary frequencies for hydrogen, the alkali–metal, alkaline–earth, and noble gas atoms. *Atom. Data Nucl. Data* **2010**, *96*, 323–331.

(17) Fedorov, D. V.; Sadhukhan, M.; Stöhr, M.; Tkatchenko, A. Quantum-Mechanical Relation between Atomic Dipole Polarizability and the van der Waals Radius. *Phys. Rev. Lett.* **2018**, *121*, 183401.

(18) Tang, K. T.; Toennies, J. P. The van der Waals potentials between all the rare gas atoms from He to Rn. *J. Chem. Phys.* **2003**, *118*, 4976–4983.

(19) Tang, K. T.; Toennies, J. P.; Wanschura, M.; Yiu, C. L. Exchange energy of alkalimetal dimer cations calculated from the atomic polarizability with the Holstein-Herring method. *Phys. Rev. A* **1992**, *46*, 3746–3752.

(20) Tao, J.; Rappe, A. M. Communication: Accurate higher-order van der Waals coefficients between molecules from a model dynamic multipole polarizability. *J. Chem. Phys.* **2016**, *144*, 031102.

(21) Gould, T.; Bučko, T. C_6 coefficients and dipole polarizabilities for all atoms and many ions in rows 1–6 of the periodic table. *J. Chem. Theory Comput.* **2016**, *12*, 3603–3613.
